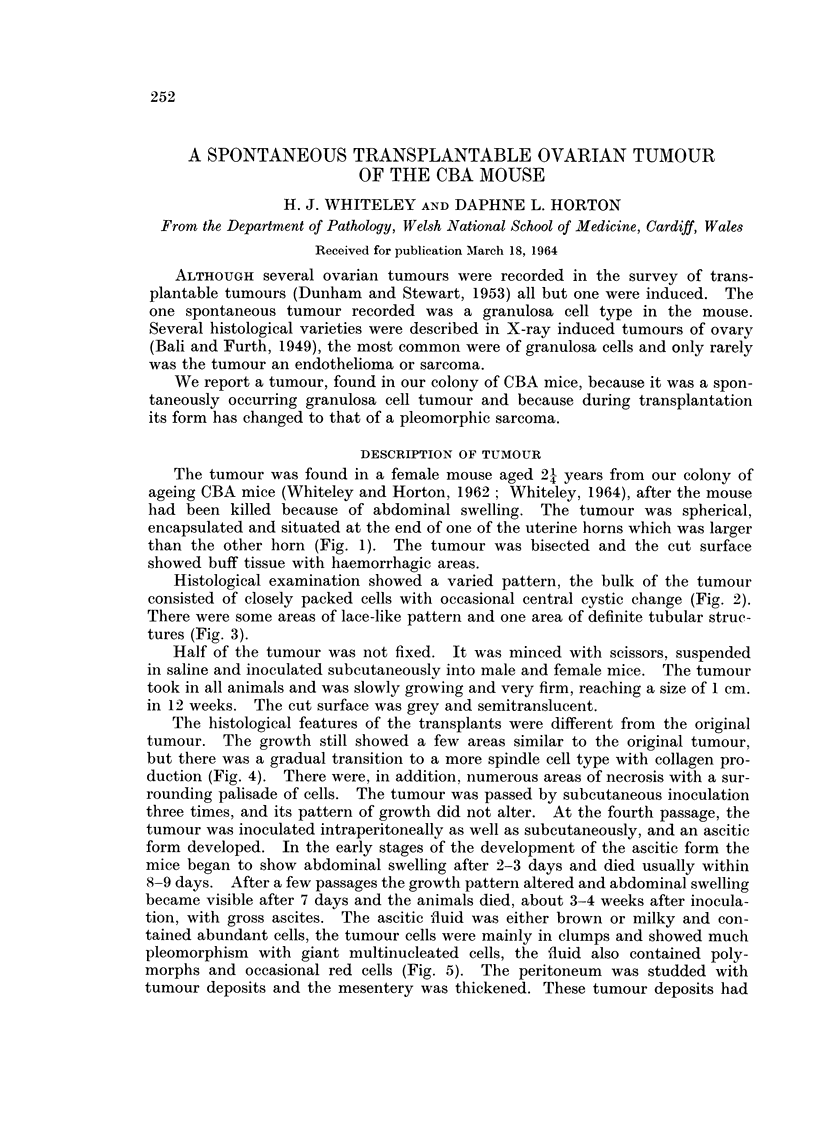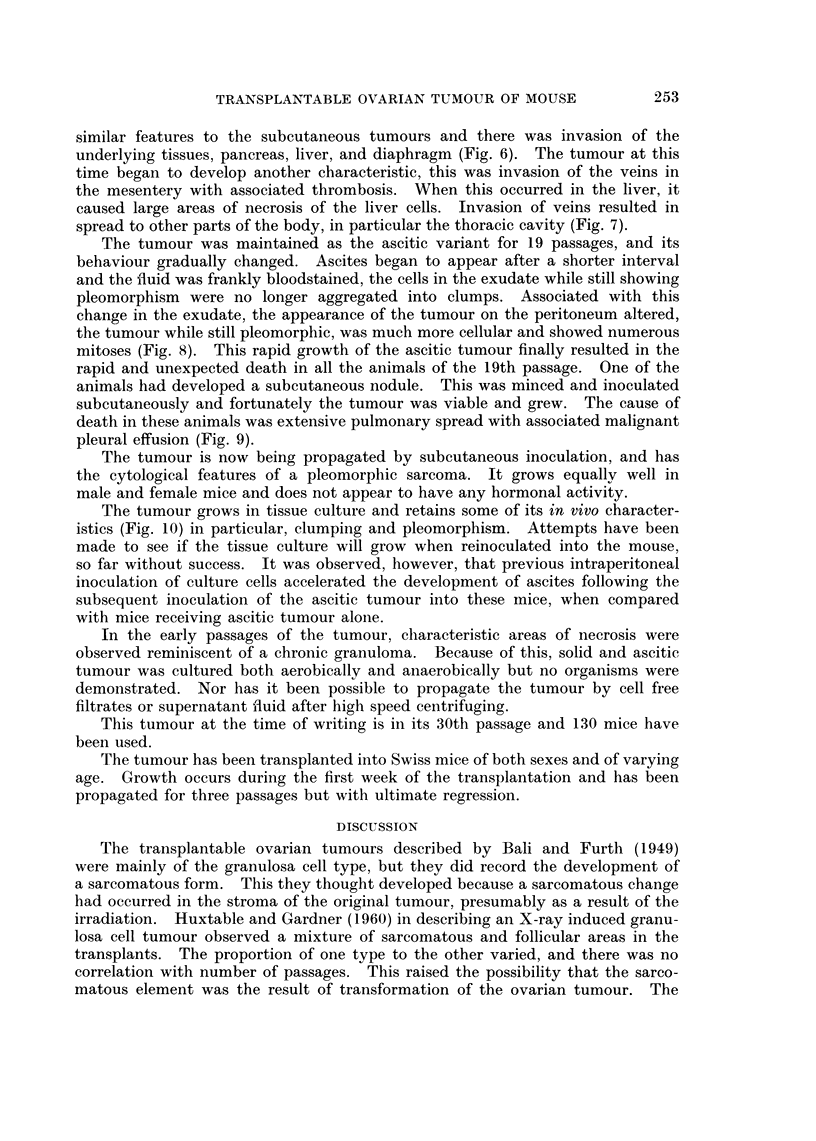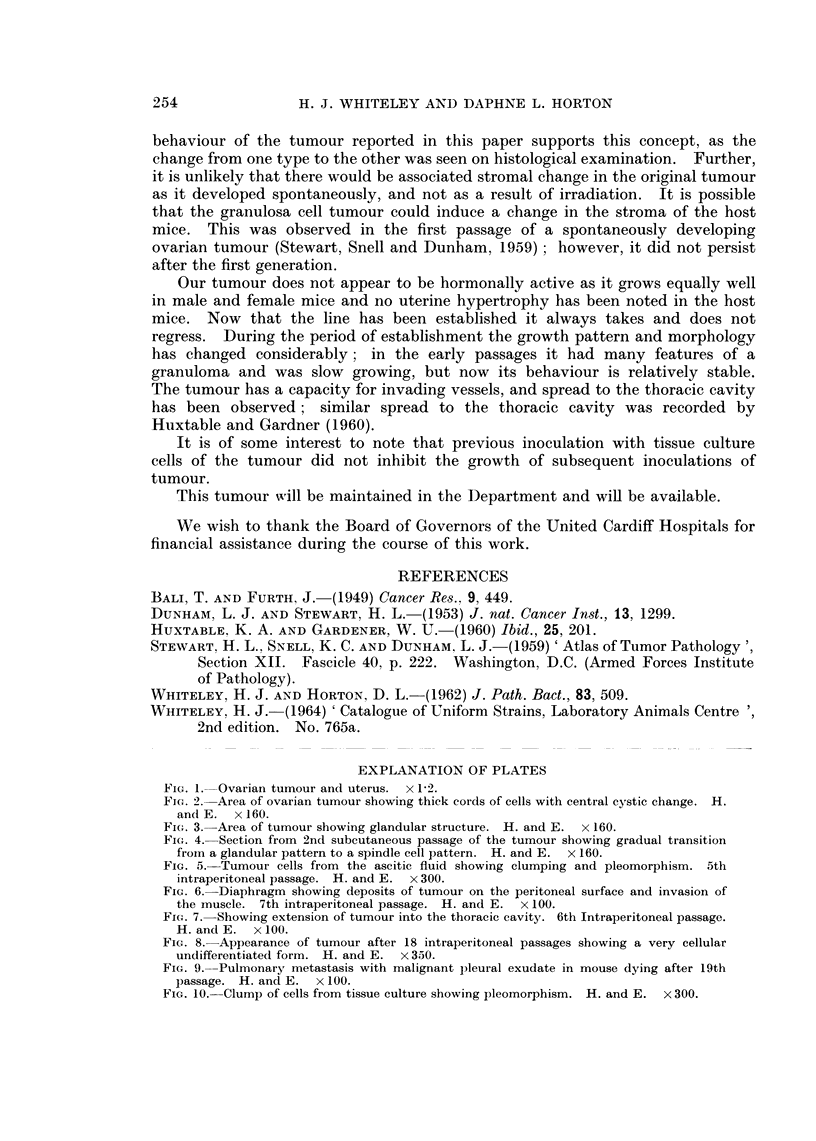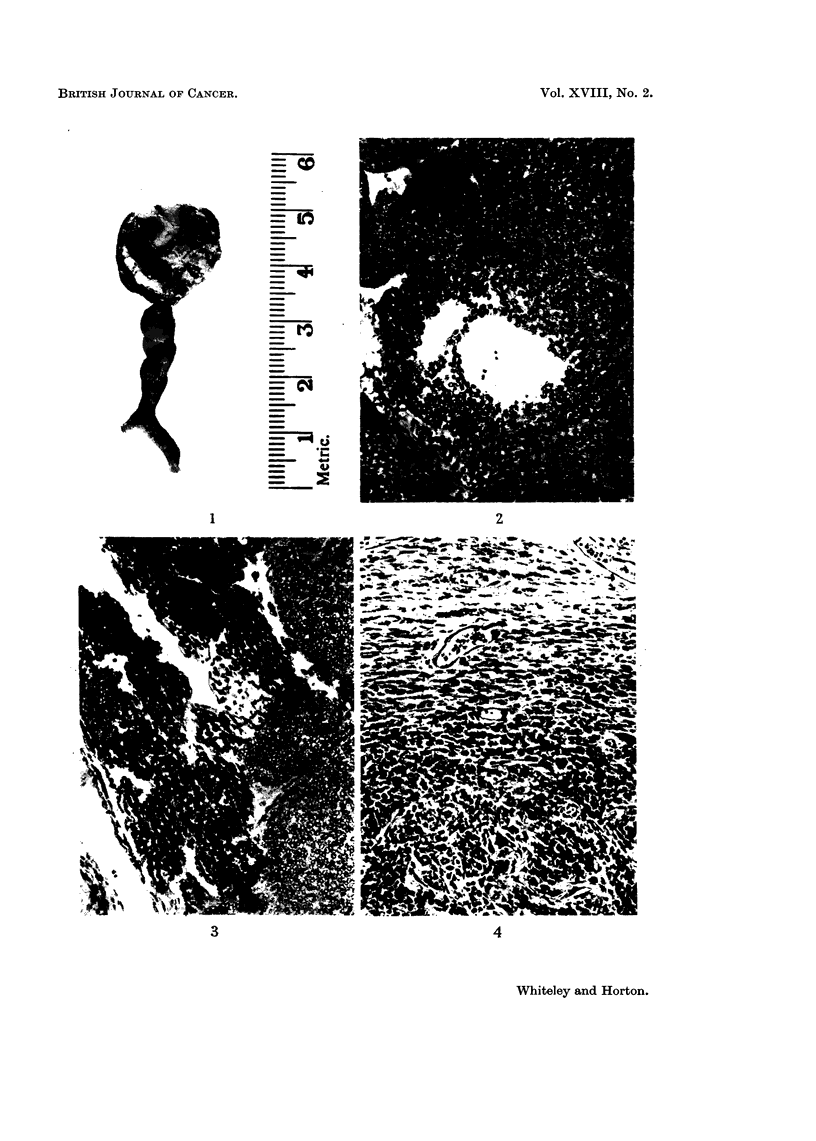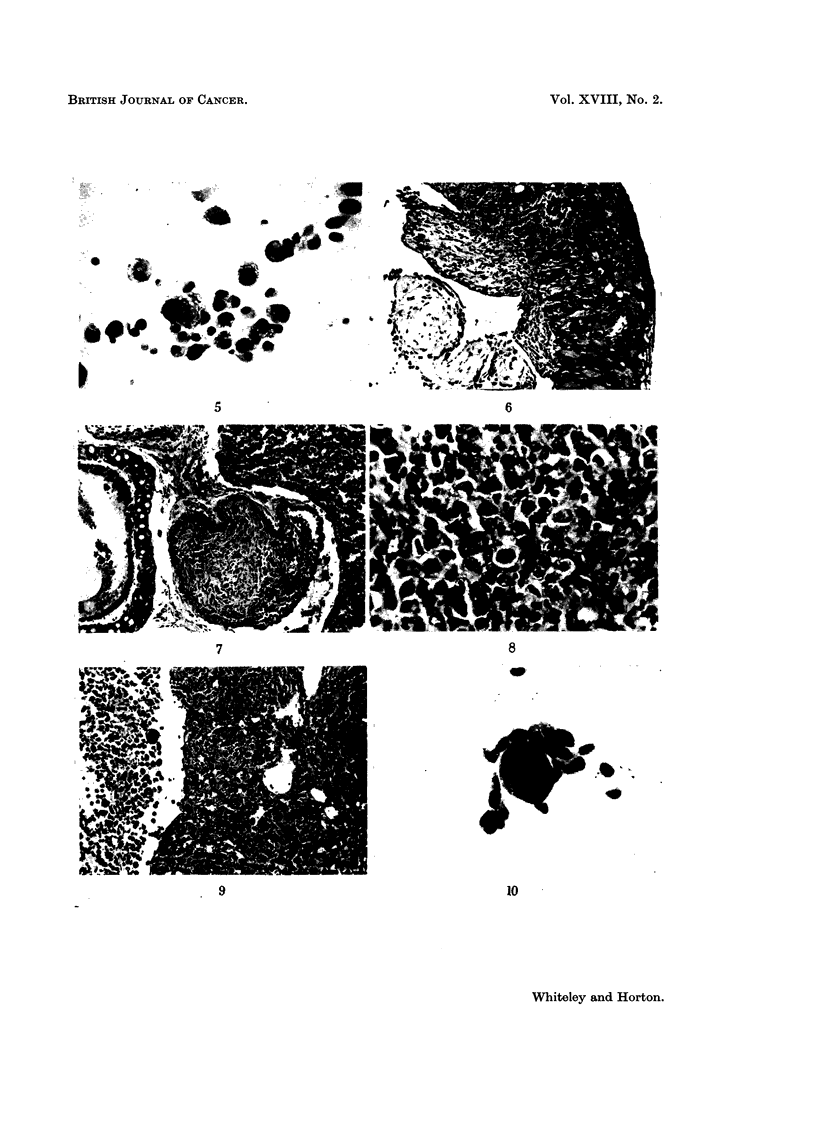# A Spontaneous Transplantable Ovarian Tumour of the CBA Mouse

**DOI:** 10.1038/bjc.1964.28

**Published:** 1964-06

**Authors:** H. J. Whiteley, Daphne L. Horton

## Abstract

**Images:**


### A SPONTANEOUS TRANSPLANTABLE OVARIAN TUMOUR OF THE CBA MOUSE

H. J. WHITELEY AND DAPHNE L. HORTON

*From the Department of Pathology, Welsh National School of Medicine, Cardiff, Wales*

Received for publication March 18, 1964

ALTHOUGH several ovarian tumours were recorded in the survey of transplantable tumours (Dunham and Stewart, 1953) all but one were induced. The one spontaneous tumour recorded was a granulosa cell type in the mouse. Several histological varieties were described in X-ray induced tumours of ovary (Bali and Furth, 1949), the most common were of granulosa cells and only rarely was the tumour an endothelioma or sarcoma.

We report a tumour, found in our colony of CBA mice, because it was a spontaneously occurring granulosa cell tumour and because during transplantation its form has changed to that of a pleomorphic sarcoma.

### DESCRIPTION OF TUMOUR

The tumour was found in a female mouse aged $2\frac{1}{4}$ years from our colony of ageing CBA mice (Whiteley and Horton, 1962 ; Whiteley, 1964), after the mouse had been killed because of abdominal swelling. The tumour was spherical, encapsulated and situated at the end of one of the uterine horns which was larger than the other horn (Fig. 1). The tumour was bisected and the cut surface showed buff tissue with haemorrhagic areas.

Histological examination showed a varied pattern, the bulk of the tumour consisted of closely packed cells with occasional central cystic change (Fig. 2). There were some areas of lace-like pattern and one area of definite tubular structures (Fig. 3).

Half of the tumour was not fixed. It was minced with scissors, suspended in saline and inoculated subcutaneously into male and female mice. The tumour took in all animals and was slowly growing and very firm, reaching a size of 1 cm. in 12 weeks. The cut surface was grey and semitranslucent.

The histological features of the transplants were different from the original tumour. The growth still showed a few areas similar to the original tumour, but there was a gradual transition to a more spindle cell type with collagen production (Fig. 4). There were, in addition, numerous areas of necrosis with a surrounding palisade of cells. The tumour was passed by subcutaneous inoculation three times, and its pattern of growth did not alter. At the fourth passage, the tumour was inoculated intraperitoneally as well as subcutaneously, and an ascitic form developed. In the early stages of the development of the ascitic form the mice began to show abdominal swelling after 2–3 days and died usually within 8–9 days. After a few passages the growth pattern altered and abdominal swelling became visible after 7 days and the animals died, about 3–4 weeks after inoculation, with gross ascites. The ascitic fluid was either brown or milky and contained abundant cells, the tumour cells were mainly in clumps and showed much pleomorphism with giant multinucleated cells, the fluid also contained polymorphs and occasional red cells (Fig. 5). The peritoneum was studded with tumour deposits and the mesentery was thickened. These tumour deposits had similar features to the subcutaneous tumours and there was invasion of the underlying tissues, pancreas, liver, and diaphragm (Fig. 6). The tumour at this time began to develop another characteristic, this was invasion of the veins in the mesentery with associated thrombosis. When this occurred in the liver, it caused large areas of necrosis of the liver cells. Invasion of veins resulted in spread to other parts of the body, in particular the thoracic cavity (Fig. 7).

The tumour was maintained as the ascitic variant for 19 passages, and its behaviour gradually changed. Ascites began to appear after a shorter interval and the fluid was frankly bloodstained, the cells in the exudate while still showing pleomorphism were no longer aggregated into clumps. Associated with this change in the exudate, the appearance of the tumour on the peritoneum altered, the tumour while still pleomorphic, was much more cellular and showed numerous mitoses (Fig. 8). This rapid growth of the ascitic tumour finally resulted in the rapid and unexpected death in all the animals of the 19th passage. One of the animals had developed a subcutaneous nodule. This was minced and inoculated subcutaneously and fortunately the tumour was viable and grew. The cause of death in these animals was extensive pulmonary spread with associated malignant pleural effusion (Fig. 9).

The tumour is now being propagated by subcutaneous inoculation, and has the cytological features of a pleomorphic sarcoma. It grows equally well in male and female mice and does not appear to have any hormonal activity.

The tumour grows in tissue culture and retains some of its *in vivo* characteristics (Fig. 10) in particular, clumping and pleomorphism. Attempts have been made to see if the tissue culture will grow when reinoculated into the mouse, so far without success. It was observed, however, that previous intraperitoneal inoculation of culture cells accelerated the development of ascites following the subsequent inoculation of the ascitic tumour into these mice, when compared with mice receiving ascitic tumour alone.

In the early passages of the tumour, characteristic areas of necrosis were observed reminiscent of a chronic granuloma. Because of this, solid and ascitic tumour was cultured both aerobically and anaerobically but no organisms were demonstrated. Nor has it been possible to propagate the tumour by cell free filtrates or supernatant fluid after high speed centrifuging.

This tumour at the time of writing is in its 30th passage and 130 mice have been used.

The tumour has been transplanted into Swiss mice of both sexes and of varying age. Growth occurs during the first week of the transplantation and has been propagated for three passages but with ultimate regression.

### DISCUSSION

The transplantable ovarian tumours described by Bali and Furth (1949) were mainly of the granulosa cell type, but they did record the development of a sarcomatous form. This they thought developed because a sarcomatous change had occurred in the stroma of the original tumour, presumably as a result of the irradiation. Huxtable and Gardner (1960) in describing an X-ray induced granulosa cell tumour observed a mixture of sarcomatous and follicular areas in the transplants. The proportion of one type to the other varied, and there was no correlation with number of passages. This raised the possibility that the sarcomatous element was the result of transformation of the ovarian tumour. The behaviour of the tumour reported in this paper supports this concept, as the change from one type to the other was seen on histological examination. Further, it is unlikely that there would be associated stromal change in the original tumour as it developed spontaneously, and not as a result of irradiation. It is possible that the granulosa cell tumour could induce a change in the stroma of the host mice. This was observed in the first passage of a spontaneously developing ovarian tumour (Stewart, Snell and Dunham, 1959) ; however, it did not persist after the first generation.

Our tumour does not appear to be hormonally active as it grows equally well in male and female mice and no uterine hypertrophy has been noted in the host mice. Now that the line has been established it always takes and does not regress. During the period of establishment the growth pattern and morphology has changed considerably ; in the early passages it had many features of a granuloma and was slow growing, but now its behaviour is relatively stable. The tumour has a capacity for invading vessels, and spread to the thoracic cavity has been observed ; similar spread to the thoracic cavity was recorded by Huxtable and Gardner (1960).

It is of some interest to note that previous inoculation with tissue culture cells of the tumour did not inhibit the growth of subsequent inoculations of tumour.

This tumour will be maintained in the Department and will be available.

We wish to thank the Board of Governors of the United Cardiff Hospitals for financial assistance during the course of this work.

### REFERENCES

Bali, T. and Furth, J.—(1949) *Cancer Res.*, **9**, 449.
Dunham, L. J. and Stewart, H. L.—(1953) *J. nat. Cancer Inst.*, **13**, 1299.
Huxtable, K. A. and Gardener, W. U.—(1960) *Ibid.*, **25**, 201.
Stewart, H. L., Snell, K. C. and Dunham, L. J.—(1959) ‘ Atlas of Tumor Pathology ’, Section XII. Fascicle 40, p. 222. Washington, D.C. (Armed Forces Institute of Pathology).
Whiteley, H. J. and Horton, D. L.—(1962) *J. Path. Bact.*, **83**, 509.
Whiteley, H. J.—(1964) ‘ Catalogue of Uniform Strains, Laboratory Animals Centre ’, 2nd edition. No. 765a.

### EXPLANATION OF PLATES

Fig. 1.—Ovarian tumour and uterus. ×1·2.
Fig. 2.—Area of ovarian tumour showing thick cords of cells with central cystic change. H. and E. ×160.
Fig. 3.—Area of tumour showing glandular structure. H. and E. ×160.
Fig. 4.—Section from 2nd subcutaneous passage of the tumour showing gradual transition from a glandular pattern to a spindle cell pattern. H. and E. ×160.
Fig. 5.—Tumour cells from the ascitic fluid showing clumping and pleomorphism. 5th intraperitoneal passage. H. and E. ×300.
Fig. 6.—Diaphragm showing deposits of tumour on the peritoneal surface and invasion of the muscle. 7th intraperitoneal passage. H. and E. ×100.
Fig. 7.—Showing extension of tumour into the thoracic cavity. 6th Intraperitoneal passage. H. and E. ×100.
Fig. 8.—Appearance of tumour after 18 intraperitoneal passages showing a very cellular undifferentiated form. H. and E. ×350.
Fig. 9.—Pulmonary metastasis with malignant pleural exudate in mouse dying after 19th passage. H. and E. ×100.
Fig. 10.—Clump of cells from tissue culture showing pleomorphism. H. and E. ×300.